# Comparison of Secondary Metabolite Extraction Methods in *Hamelia patens* Jacq. and Their Inhibitory Effect on *Fusarium oxysporum* f. sp. *radicis-lycopersici*

**DOI:** 10.3390/metabo15010023

**Published:** 2025-01-06

**Authors:** Daniel Jafet Valle Ortiz, Dolores Guadalupe Aguila Muñoz, María del Carmen Cruz López, Diana Verónica Cortés Espinosa, Martha Rosales Castro, Fabiola Eloísa Jiménez Montejo

**Affiliations:** 1 Centro de Investigación en Biotecnología Aplicada, Instituto Politécnico Nacional, Ex-Hacienda San Juan Molino Carretera Estatal Tecuexcomac-Tepetitla Km 1.5, Tlaxcala C.P. 90700, Mexico; dvalleo2100@alumno.ipn.mx (D.J.V.O.); daguilam1800@ipn.mx (D.G.A.M.); ccruzl@ipn.mx (M.d.C.C.L.); dcortes@ipn.mx (D.V.C.E.); 2 Centro Interdisciplinario de Investigación para el Desarrollo Integral Regional Unidad Durango, Instituto Politécnico Nacional, Calle Sigma Núm. 119 Fracc. 20 de noviembre, Durango, Durango C.P. 34220, Mexico; mrosalesc@ipn.mx

**Keywords:** *Hamelia patens* Jacq., *Fusarium oxysporum* f. sp. *radicis-lycopersici*, phytochemical profile, antifungal activity, UPLC-MS

## Abstract

**Background**: *Hamelia patens* Jacq. (HP) is widely recognized in traditional medicine for its antimicrobial properties, which are attributed to secondary metabolites such as phenolic compounds, alkaloids, and terpenes. *Fusarium oxysporum* f. sp. *radicis-lycopersici* (Fo), a phytopathogenic fungus affecting economically important crops, is managed with fungicides like benzimidazoles and azoles. Excessive use of these compounds has led to resistance and environmental contamination, highlighting the need for sustainable alternatives. This study aimed to optimize the extraction of secondary metabolites from HP leaves and flowers, evaluate their antifungal activity, and assess the impact of extraction methods and plant parts on chemical composition and efficacy. **Methods**: Three extraction methods were employed: consecutive maceration (CM) using solvents of ascending polarity; total maceration (TM), which is a single-step methanol-based method; and ultrasound-assisted maceration (UAM) employing ultrasonic waves with methanol. Extracts were characterized by quantifying total phenols (TP), condensed tannins (TC), flavonoids (Fl), alkaloids (TA), sterols (TS), and saponins (S) using colorimetric assays and UPLC-MS. Multivariate analyses, including PCA, PLS-DA, OPLS-DA, and Pearson correlation, evaluated the relationships between the chemical profiles and antifungal activity. **Results**: Leaf extracts exhibited higher flavonoid and tannin contents than flower extracts. CMML showed the highest antifungal activity (IC_50_ 3.7% *w*/*v*), which was associated with elevated levels of these compounds. Significant correlations linked antifungal activity with rutin (HP21) and kaempferol-3-O-β-rutinoside (HP29). **Conclusions**: Methanolic extracts of HP exhibited significant antifungal activity against Fo. These findings highlight the importance of optimizing extraction methods and selecting specific plant parts to enhance bioactive compound efficacy, offering a sustainable approach to pathogen management.

## 1. Introduction

*Hamelia patens* Jacq. is a perennial shrub of the Rubiaceae family, which is commonly known as “bayetilla” or firebush [1]. It is native to Latin America and grows in tropical and subtropical regions ranging from Mexico to Argentina [2]. HP has been traditionally used in medicine for its analgesic, anti-inflammatory, antimicrobial, and wound-healing properties, which are attributed to the presence of secondary metabolites [3,4], including alkaloids, terpenes, and phenolic compounds such as flavonoids, tannins, and phenolic acids [5]. The diversity and concentration of these metabolites vary depending on the plant organ (leaves, flowers, or roots) and the extraction method used, such as maceration, Soxhlet extraction, sonication, or microwave-assisted extraction (MAE) [6,7]. Each extraction method presents specific advantages and limitations that affect the quantity and quality of the extracted compounds [8,9]. It has been shown that ethanolic and methanolic extracts of the aerial parts of *H. patens* obtained through maceration exhibit a greater diversity of secondary metabolites than other solvents [10]. On the other hand, sonication has proven particularly effective in preserving thermosensitive compounds [11]. MAE, in turn, offers notable advantages such as reduced extraction time, energy efficiency, and enhanced yields of thermosensitive compounds [12].

Several researchers have investigated the antimicrobial activity of HP extracts obtained through various extraction methods. For instance, Megala et al. reported that methanolic extracts of HP leaves and flowers produced inhibition zones of 14 mm against *Staphylococcus aureus*, which were attributed to phenolic compounds [13]. Furthermore, flavonoids and tannins have been shown to inhibit microbial and viral replication [14].

Despite these advances, the antifungal potential of HP against phytopathogenic fungi remains underexplored. *Fusarium oxysporum* f. sp. *radicis-lycopersici* is one of the most prominent phytopathogens, which affects more than 100 species of commercially important plants, causing vascular diseases that threaten global agricultural production [15]. This fungus is commonly controlled with chemical fungicides, which lead to resistances that can have detrimental effects on the environment and human health [16]. These challenges underscore the urgent need to develop sustainable and effective alternatives for its control.

The present study addresses these limitations by evaluating how three extraction methods (consecutive maceration, total maceration, and ultrasound-assisted maceration) affect the content of bioactive secondary metabolites in the leaves and flowers of HP. Additionally, it examines the correlation between these metabolites and their antifungal activity against *Fo*. This study contributes to the development of sustainable strategies for managing phytopathogenic fungi and expands the understanding of how extraction methods influence the chemical composition of HP, filling existing gaps in the literature and offering potential applications in agricultural biotechnology.

## 2. Materials and Methods

### 2.1. Reagents

All reagents and standards were commercially available from Sigma-Aldrich^®^ (Sigma-Aldrich Co., St. Louis, MO, USA). Solvents were dried and freshly distilled before use. Chromatographic solvents were grade LC-MS reagents from J.T. Baker^®^ (Avantor Performance Materials, Phillipsburg, NJ, USA). Leucine-enkephalin (*m*/*z*) was used as the reference lock mass and was commercially available from Waters^®^ (Waters Corporation, Milford, MA, USA).

### 2.2. Collection of Plant Material

Leaves (L) and flowers (F) of HP were collected in Cuetzalan, located in Puebla, Mexico (Latitude 20.0184890; Longitude −97.5173427), in January 2023. The samples were dried at 25 °C for 10 days, ground into a fine powder, and stored at room temperature in sealed containers until further processing.

### 2.3. Obtaining Extracts

The extracts were obtained by CM, TM, and UAM. Both the consecutive maceration and the total maceration were carried out at room temperature (20 ± 2 °C). Ten grams of plant material were weighed for each extraction method, and 100 mL of solvent was used. The macerations TM and CM were carried out for 4 days with solvent change every 24 h. In CM, solvents of ascending polarity, hexane (H), methylene chloride (D), and methanol (M) were used to favor the selective extraction of low-, medium-, and high-polarity compounds. The resulting treatments were named hexane in flowers (CMHF), hexane in leaves (CMHL), dichloromethane in flowers (CMDF), dichloromethane in leaves (CMDL), methanol in flowers (CMMF), and methanol in leaves (CMML). In TM, only methanol was used, and the treatments were identified as methanol in flowers (TMF) and methanol in leaves (TML). In UAM, the samples were subjected to ultrasound for 30 min at 40 kHz and 280 W (Branson 8800 Ultrasonic cleaner, Branson Ultrasonics Corporation, Brookfield, CT, USS, 25 L, at 25 ± 2 °C). The extracts obtained by ultrasound were methanol flowers (UAMF) and methanol leaves (UAML). The excess solvent was removed employing a vacuum rotary evaporator (Büchi® Rotavapor, Büchi Labortechnik AG, Flawil, Switzerland) at 40 °C. The extracts obtained were dried, lyophilized, and stored at −20 °C for conservation and later use. Extraction yields were calculated as follows:

$$\mathrm{Yield}\;\left(\%\frac{w}{w}\right)=\left(\frac{Dry\;extract\;weight}{Dry\;sample\;weight}\right)\times 100$$

### 2.4. Phytochemical Profile of the Extracts

The total phenolic content was measured using the Folin–Ciocalteu method [17] with certain modifications. The reaction mixture was prepared with 0.2 mL extract (5 mg/mL), 2 mL solution A (2% Na_2_CO_3_, 1% CuSO_4_, and 2.7% KNaC_4_H_4_O_6_ 4H_2_O), and 0.4 mL of NaOH (0.5 N). Subsequently, 0.2 mL of the Folin–Ciocalteu solution (1:1) was added, and the mixture was allowed to stand for 30 min at room temperature. The absorbance was measured at 750 nm. The total phenolic content was expressed as milligrams equivalent of gallic acid per gram of dry extract (meq-AG/g). Total flavonoids were evaluated using the AlCl_3_ method with slight modifications [17]. Amounts of 0.1 mL of extract (5 mg/mL), 0.30 mL of absolute ethanol, 0.02 mL of AlCl_3_ (10%), 0.02 mL of CHCOOK, and 0.56 mL of distilled water were mixed. The mixture stood for 30 min at room temperature, and the reading was taken at 415 nm. The flavonoid content was determined with a quercetin calibration curve and expressed as mg of quercetin equivalents per gram of dry extract (meq-Q/g). The tannin content was determined using the vanillin/HCl method [18]. A total of 2 mL of extract (25 mg/mL) was placed in a tube and heated in a water bath at 30 °C for 20 min; 0.4 mL of this sample was removed, and then 2 mL of vanillin solution (1% in methanol) was added. The tube was placed again in a water bath at 30 °C for 20 min. Finally, the reading was done at 550 nm, and the tannin content was expressed as mg of catechin per gram of dry extract (meq-C/g). Total saponins were determined by the vanillin-H_2_SO_4_ assay [19]. A total of 0.125 mL of the extract (5 mg/mL), 0.125 mL of 8% vanillin, and 1.25 mL of H_2_SO_4_ were mixed and heated in a water bath at 60 °C for 10 min. After cooling in an ice bath for 5 min, the samples were measured at 560 nm. The saponin content was expressed as mg of diosgenin equivalents (DE) per g of dry extract (meq-D/g). Total alkaloids were quantified using the method based on the reaction with bromocresol green [20]. Five mg of extract was dissolved in 1 mL of HCl (2N), vortexed, centrifuged, and filtered. Then, 1 mL of the extract was washed with chloroform (3 × 2 mL), and the chloroform phase was adjusted to neutral pH with 0.1 N NaOH. This was mixed with 5 mL of bromocresol green and phosphate buffer (pH 4.7), washed again with chloroform (3 × 3.5 mL), and the absorbance was measured at 470 nm. The results were expressed as mg of gramine equivalents per g of dry extract (meq-G/g). Finally, total sterols were measured with the Liebermann–Bürchard test [15]. A total of 25 mg of sample was dissolved in 5 mL of chloroform–methanol (2:1), vortexed, heated to 50 °C for 10 min, centrifuged, and filtered. A total of 0.5 mL of saturated CaCl_2_ solution was added to the filtrate, two phases were separated, and the lower phase was kept, which was diluted with chloroform/methanol/water (2:50:50). Finally, 0.2 mL of the extract was mixed with 2 mL of the Liebermann–Bürchard reagent and incubated at 35 °C for 10 min. The absorbance was measured at 550 nm. The results were expressed as mg cholesterol equivalents per g of dry extract (meq-Col/g).

### 2.5. Microbial Strain and Inoculum Preparation

*Fusarium oxysporum* f. sp. *radicis-lycopersici* was isolated from tomato plants exhibiting typical symptoms of *Fusarium* wilt and identified with the help of Centro Interdisciplinario de Investigación para el Desarrollo Integral Regional Unidad Sinaloa belonging to the Instituto Politécnico Nacional (CIIDIR-Sinaloa-IPN). The micro-organism was propagated in Petri dishes with potato dextrose agar (PDA) culture medium and incubated at 28 °C for 10 days.

### 2.6. Antifungal Activity

#### 2.6.1. Evaluation of Radial Growth

The assay was carried out using the plate dilution method [21]. PDA was mixed with different extract concentrations (1, 2, 3, 4, and 5%) using 1% *v*/*v* dimethyl sulfoxide (DMSO) as an adjuvant in the solubilization of the extracts. A 4 mm diameter disk of five-day-old Fo culture was placed in the center of each plate. The inoculated plates were incubated at 28 °C for 5 days. The radial growth of the fungus was measured every 24 h. After the incubation period, the antifungal effect of each treatment was calculated using the following formula:

$$\mathrm{Growth}\;\mathrm{inhibition}\;\left(\%\right)=\left(\frac{\mathrm{Growth}\;\mathrm{in}\;\mathrm{control}-\;\mathrm{Growth}\;\mathrm{in}\;\mathrm{treatment}\;}{\mathrm{Growth}\;\mathrm{in}\;\mathrm{control}}\right)\times 100$$

#### 2.6.2. Determination of Median Inhibitory Concentrations (IC_50_)

Once the percentages of inhibition of Fo radial growth were calculated, and the median inhibitory concentration (IC_50_) values were determined using the following formula:

$$IC_{50}=C_{b}+\frac{\left(50-I_{b}\right)\times \left(C_{a}-C_{b}\right)}{I_{a}-I_{b}}$$

*C_b_* and *C_a_* are concentrations below and above 50% inhibition, and *I_b_* and *I_a_* are the inhibition percentages corresponding to those concentrations [22].

### 2.7. Analysis of Extracts by UPLC-MS

Samples were analyzed using an Acquity I UPLC system (Waters Corporation, Milford, MA, USA) with a binary solvent system and autosampler coupled to a Xevo G2-XS QTOF mass spectrometer detector. The chromatographic and mass spectral components were analyzed with the Natural Products Application Solution within Waters’ UNIFI Scientific Information System version 1.9 (Waters Corporation, Milford, MA, USA). In addition, the elements were identified by verifying that the fragmentation patterns fell within the allowable error range of ±5 ppm. If the fragment ion pattern did not match, it was finally identified using a ChemSpider^®^ online database (Royal Society of Chemistry, Cambridge, UK). For the identified compounds, the matched compound generating the predicted piece from the structure was performed using the filter: Isotope match Intensity RMS Percent ≥ 20; Mass peak resolution ≥ 7000; Mass error (mDa) ≤ 2. The negative adduct was −H.

UPLC-MS analyzed different extracts using UNIFI data acquisition and processing software version 1.9 (Waters Corporation, Milford, MA, USA). We used the ACQUITY UPLC-BEH C18 column (2.1 mm × 100 mm, 1.7 μm; Waters Corporation, Manchester, UK) at 45 °C. Acetonitrile was used as mobile phase A, and water with 0.05% formic acid was used as mobile phase B. The flow rate was 0.5 mL/min, and an injection volume of 2 μL was used. A mixture of water–acetonitrile, 90:10, was used as the weak wash solvent, and water–acetonitrile, 50:50, was used as the strong wash solvent for needle wash. The elution gradient initial was 5% of A, 10% A from 0 to 2 min, 10–15% A from 2 to 5 min, 25% A from 5 to 8 min, 33% A from 8 to 12 min, 53% A from 12 to 15 min, 95% A from 15 to 18 min, 95% A from 18 to 20 min, and 95–5% A from 20 to 22 min. MS experiments were performed on a Waters Xevo G2-XS QTOF mass spectrometer (Waters Corporation, Milford, MA, USA) connected to the UPLC system through an electrospray ionization (ESI, negative) interface. API negative ion and sensitivity analyzer mode were selected for QTOF/MSE data acquisition. To obtain accurate mass precursor and fragment ion data, a mass range of *m*/*z* 100–1200 was chosen for the accurate mass precursor and fragment ion data.

The operating parameters were as follows: Mode—MSE; cone mode—specific; capillary voltage—2.5 kV; cone—40 V; cone voltage—35 V; cone gas flow—50 L/h; source temperature—100 °C; desolvation temperature—550 °C and desolvation gas flow—1000 L/h. The trap collision energy of the low-energy function was set at 6 eV, and the slope trap collision energy of the high-energy function was set at 20–60 eV (ESI−). The calibration substance was a sodium formate solution. To ensure the mass accuracy and reproducibility of the optimized MS condition, leucine–enkephalin (*m*/*z*) 554.2615 in downbeat mode was used as the reference lock mass at a concentration of 200 pg/mL and a flow rate of 10 μL/min and was sprayed into the MS instrument every 10 s.

### 2.8. Statistical Analysis

The UPLC-MS liquid chromatography data were analyzed using multivariate techniques with SIMCA software version 14.1 (Sartorius Stedim Data Analytics, Malmö, Sweden). To assess the variability in secondary metabolite content among HP extracts, principal component analysis (PCA) was performed to explore the overall distribution of the samples based on their phytochemical profiles. This was complemented with partial least squares discriminant analysis (PLS-DA) to correlate the identified compounds with the observed biological effect in *Fo*. Additionally, orthogonal partial least squares discriminant analysis (OPLS-DA) enhanced data interpretability by distinguishing predictive components from orthogonal variations, facilitating clearer identification of the metabolites responsible for antifungal activity. Pearson evaluation analysis was performed using Minitab 19^®^ statistical software (Minitab LLC, State College, PA, USA) to complement the PLS-DA and OPLS-DA results, evaluating the relationship between the overall phytochemical profile and IC_50_ values and the measurement between the identified secondary metabolites and IC_50_ values. The phytochemical profile and antifungal effect of the extracts were evaluated with a completely randomized experimental design using analysis of variance (ANOVA) and comparison of means with the Tukey method (*p* = 0.05) in RStudio (version 4.4.1; RStudio, PBC, Boston, MA, USA). Each experiment was carried out in quadruplicate (n = 4).

## 3. Results

### 3.1. Extract Yields

Table 1 demonstrates significant variations in the yield of HP extracts depending on the plant part utilized and the extraction method applied. Leaf extracts exhibited the highest yields, with TML achieving the highest extraction efficiency (13.1%), followed by UAML (11.4%) and CMML (8.5%). In contrast, the flower extracts showed lower yields, with the TMF yielding the highest percentage (4.8%), followed closely by the UAMF (4.6%) and CMMF (4.4%). These differences may be attributed to a higher concentration of polar and soluble metabolites in the leaves, as well as the superior efficiency of the total maceration method in recovering these compounds compared to other techniques. Conversely, low yields were observed in extracts obtained using medium-polarity (CMDL and CMDF) and low-polarity solvents (CMHL and CMHF), with percentages ranging between 0.2% and 1.2%. This suggests that the majority of the metabolites in HP exhibit polar characteristics, highlighting the importance of solvent polarity and extraction techniques in optimizing metabolite recovery.

### 3.2. Phytochemical Profile Analysis of the Extracts

The phytochemical compositions of the extracts, summarized in Table 2, revealed significant variations in the secondary metabolite concentrations depending on the extraction method used. Ultrasound-assisted extracts, UAML and UAMF, exhibited the highest total phenol concentrations (203.8 and 175.9 meq-AG/g, respectively), while the lowest concentration was found in the CMHL (30.7 meq-AG/g). Flavonoid content was notably higher in the TML (32.4 meq-Q/g) and UAML (31.9 meq-Q/g), significantly exceeding the concentrations detected in the CMHF (3.4 meq-Q/g) and CMHL (5.1 meq-Q/g). Condensed tannins were predominantly present in the CMML (47.3 meq-C/g) and UAML (39.4 meq-C/g), while they were undetectable in extracts obtained using solvents of medium- and low-polarity (CMHL, CMDL, CMHF, and CMDF). Saponins were most abundant in the UAMF (1028.2 meq-D/g), followed by the UAML (967.5 meq-D/g) and CMDL (585.5 meq-D/g). The alkaloid content was generally low and only detectable in select extracts, with the highest concentration found in the UAML (0.5 meq-G/g). Finally, sterols were most concentrated in the UAML (60.4 meq-Col/g) and UAMF (56.5 meq-Col/g), whereas the TML and TMF exhibited the lowest sterol levels, with 1.8 and 1.7 meq-Col/g, respectively.

### 3.3. Antifungal Effect of the Extracts

**Figure 1:**
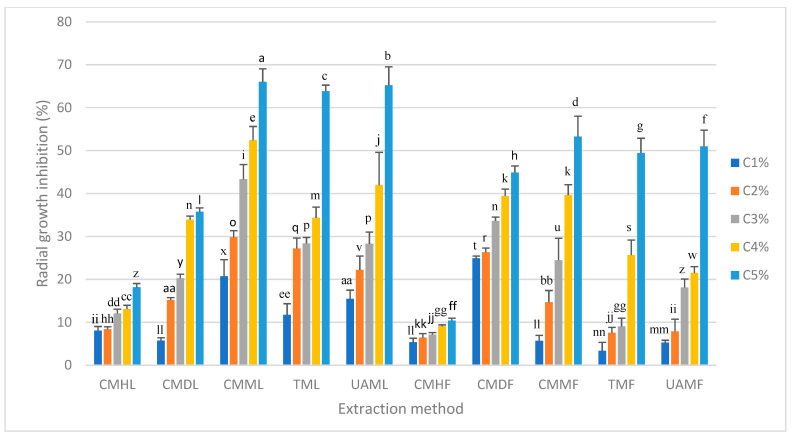
Antifungal effect with different concentrations of extracts (1–5% *w*/*v*) of *H*. *patens* on *Fusarium oxysporum* f. sp. *radicis-lycopersici*. C1%: 1% extract concentration; C2%: 2% extract concentration; C3%: 3% extract concentration; C4%: 4% extract concentration; C5%: 5% extract concentration. Different letters indicate significant differences α = 0.05 between treatments. Data are expressed as % inhibition ± standard deviation, where n = 4.

The antifungal activity of the HP extracts (1–5% *w*/*v*) was evaluated by measuring the radial growth of Fo. All extracts demonstrated antifungal properties, exhibiting a dose-dependent effect, wherein higher extract concentrations significantly enhanced fungal inhibition (Figure 1). Methanolic extracts derived from flowers exhibited moderate inhibitory effects, achieving inhibition percentages between 49% and 53% at the maximum concentration of 5%. This suggests that the compounds present in the flowers exert a limited effect on fungal growth.

In contrast, methanolic extracts obtained from leaves demonstrated greater inhibitory effects, with inhibition rates ranging from 64% to 66% at the same concentration. The calculation of IC_50_ values (Table 3) revealed that leaf extracts had the lowest IC_50_ values, indicating higher antifungal efficacy compared to flower extracts. Notably, the consecutive methanolic maceration of leaves (CMML) displayed the lowest IC_50_ value of 3.7%, suggesting a higher concentration or greater activity of the secondary metabolites responsible for Fo inhibition. Conversely, extracts obtained with non-polar solvents, such as hexane and dichloromethane, did not achieve significant inhibition percentages to allow the calculation of IC_50_ values within the evaluated concentration range.

### 3.4. Correlation of the Antifungal Effect with the Photochemical Profile of H. patens

The Pearson correlation analysis between the phytochemical profile of the extracts and their IC_50_ values against Fo (Table 4) revealed that condensed tannins (r = 0.902), total phenols (r = 0.735), and total alkaloids (r = 0.686) exhibited the strongest correlations with antifungal activity. These findings suggest that these metabolites play a primary role in the inhibitory effect observed. The positive correlation with IC_50_ indicates that higher concentrations of these metabolites are associated with enhanced antifungal efficacy of the extracts. In contrast, saponins, sterols, and flavonoids displayed negative correlations, suggesting that their contribution to the antifungal activity may be limited or secondary in nature.

### 3.5. Identification of Compounds in H. patens Extracts

**Figure 2:**
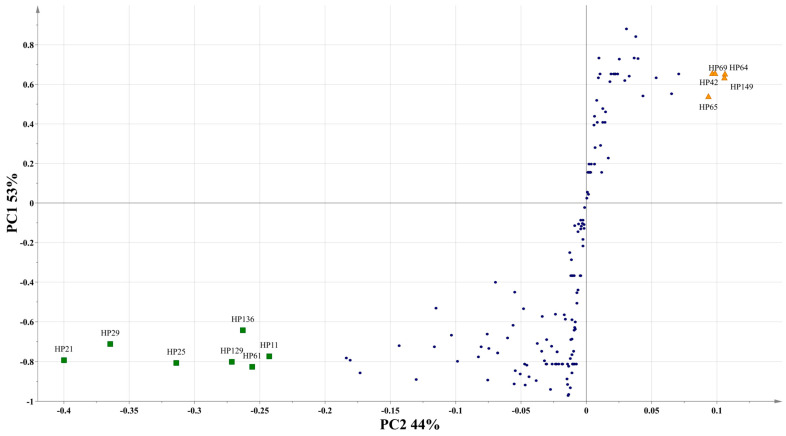
S-chart of the comparison of the phytochemical profile of the secondary metabolites found in leaf and flower extracts of *H. patens*; 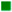 Most abundant compounds in leaves; 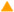 Most abundant compounds in flowers.

After evaluating the antifungal activity across all extracts, only the methanolic extracts were subjected to analysis via UPLC-MS. Hexane and dichloromethane extracts were excluded from this analysis due to their minimal antifungal efficacy, as they did not surpass 50% inhibition at the concentrations tested. One hundred and sixty-one secondary metabolites were identified across the methanolic extracts (Table 5). Chromatograms of UPLC of each of the extracts are shown in Figures S1–S6 in the supplementary material. In leaf-derived extracts, 100 metabolites were detected in CMML, 92 in TML, and 87 in UAML. Conversely, 88 metabolites were identified in CMMF, 85 in TMF, and 83 in UAMF in flower-derived extracts. Among the identified secondary metabolites, 42 flavonoids, 28 phenolic acids, 42 glycosylated phenols, and nine tannins stood out. Through the S-chart (Figure 2), it was found that in leaves, there was a greater abundance of flavonoids such as rutin (HP21), kaempferol-3-O-β-rutinoside (HP29), and 7-O-α-L-rhamnosyl-3-O-β-D-glucopyranosyl kaempferol (HP25), and tannins, including procyanidin B1 (HP129) and cinchonain Ia (HP136). In contrast, in flowers, a greater diversity of phenolic acids was found, highlighting terminolic acid (HP69), 1,3-O-dicaffeoylquinic acid (HP64), and 1,5-O-dicaffeoylquinic acid (HP65).

### 3.6. Clustering of Extracts by PCA

**Figure 3:**
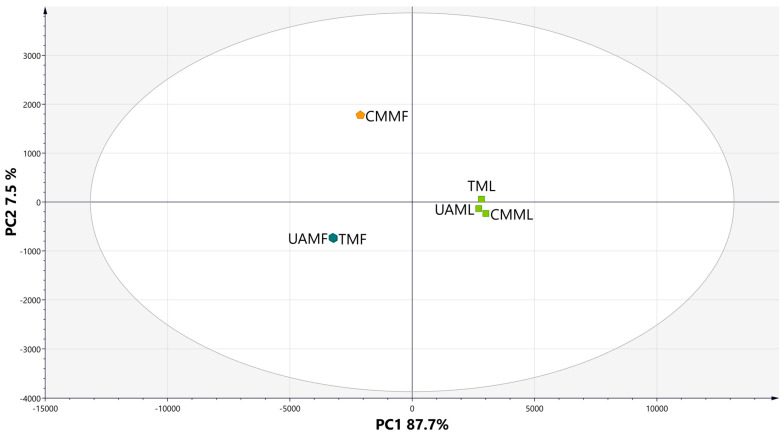
PCA scores graph of *H*. *patens* extracts.

A principal component analysis (PCA) was performed to classify the extracts based on their phytochemical profiles, enabling the visualization of similarities and differences among them. The analysis identified distinct patterns influenced by the type of plant tissue (leaves or flowers) and the extraction method employed (Figure 3). Three well-defined clusters were observed: Cluster 1, comprising CMMF; Cluster 2, including UAMF and TMF; and Cluster 3, consisting of TML, UAML, and CMML. The validity of the method was supported by an accumulated variance (R^2^X) of 98.5%, which was distributed across two principal components: PC1, which accounted for 87.7% of the variance, and PC2, which explained an additional 7.59%.

**Figure 4:**
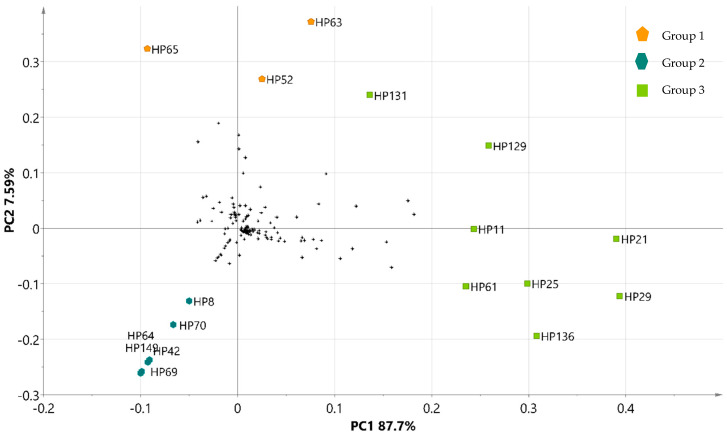
Loading plot of secondary metabolites on the principal components (PCA).

Rutin (HP21) was the predominant metabolite across all extracts; however, other compounds were also present in all of them, with higher concentrations observed in the leaf extracts. These included kaempferol-3-O-β-rutinoside (HP29), 7-O-α-L-rhamnosyl-3-O-β-D-glucopyranosyl kaempferol (HP25), 5-O-feruloylquinic acid (HP61), leufolin A (HP11), procyanidin B1 (HP129), and arecatannin A1 (HP131), which were localized in quadrants 1 and 4 of the loading plot (Figure 4), corresponding to the position of group 3. The metabolites 7-O-[4′-O-(3″,4″-dihydroxycinnamyl)-β-D-glucopyranosyl]-6-methoxycoumarin (HP63), 4-O-caffeoylquinic acid (HP52), and 1,5-O-dicaffeoylquinic acid (HP65) were more abundant in CMMF (group 1), while compounds exclusive to group 2, such as 1,3-O-dicaffeoylquinic acid (HP64), elculentic acid (HP149), terminolic acid (HP69), spinogenin C7 (HP42), nevadensin-7-O-[α-L-rhamnosyl(1→6)]-β-D-glucoside (HP8), and 2-hydroxyesculentic acid (HP70), were predominantly located in the third quadrant.

### 3.7. Partial Least Squares Analysis (PLS)

**Figure 5:**
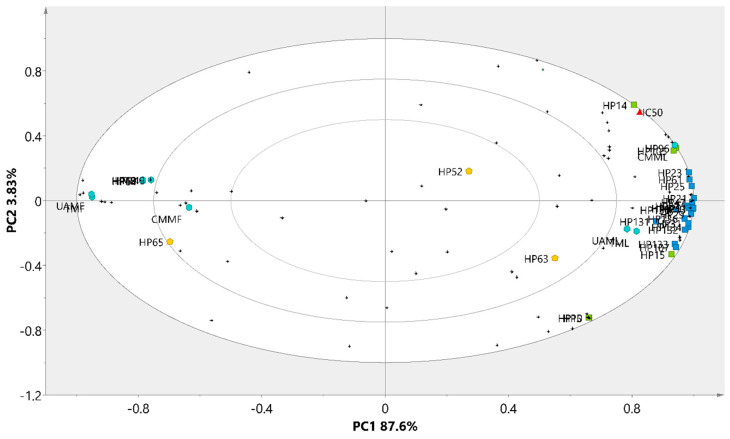
PLS-DA Biplot of HP extracts concerning antifungal activity (IC_50_) and identified phytochemical compounds. 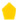 Metabolites present in group 1; 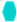 Metabolites present in group 2; 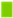 Metabolites present in group 3; 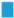 Metabolites present in all extracts but greater abundance in leaves.

PLS analysis facilitated the integration of PCA-derived data with antifungal efficacy, as measured by IC_50_ values, providing a more comprehensive understanding of the relationship between bioactive compounds and Fo inhibition. In the PLS biplot (Figure 5), compounds located furthest from the origin were directly correlated with extracts exhibiting the highest inhibitory activity against the fungus. This correlation was further validated by interpreting the “Variables of Importance for Projection” (VIP) scores and Pearson correlation, highlighting the compounds most influential in the PLS model. Compounds with a VIP score greater than 0.9 were identified as the primary contributors to the PLS model (Table 6). Among the most prominent compounds, rutin (HP21), kaempferol-3-O-β-rutinoside (HP29), 7-O-α-L-rhamnosyl-3-O-β-D-glucopyranosyl kaempferol (HP25), procyanidin B1 (HP129), leufolin A (HP11), and 5-O-feruloylquinic acid (HP61) stood out. Additionally, (-)-epicatechin (HP13), chlorogenic acid (HP47), quercetin-3-O-β-D-glucopyranoside (HP23), cinchonain B (HP133), procyanidin C2 (HP132), kaempferol-3-O-β-D-glucopyranosyl-(1→2)-β-D-galactopyranosyl-(1→2)-β-D-glucopyranoside (HP9), 6-O-galloyl-glucose (HP73), 2-hydroxyesculentic acid (HP70), menthoside (HP117), and macranthion G (HP105) were identified. These compounds, present in all extracts, were also highlighted in the PCA analysis.

Upon analyzing the Pearson correlation (PC) values, several compounds exhibited a strong positive correlation with antifungal activity. Quercetin-3-O-β-D-glucopyranoside (HP23) showed a PC value of 0.905, helonioside B (HP96) had a value of 0.945, and tectoruside (HP14) reached a value of 0.982. These high PC values indicate a robust association between these compounds and the inhibition of *Fo*, contributing to a reduction in IC_50_ values and enhancing the bioactivity of the extracts.

Regarding efficacy, the CMML, UAML, and TML extracts, which contain significant concentrations of these highly correlated compounds and have elevated VIP scores, exhibited the highest antifungal activity. This suggests that compounds such as rutin (HP21), kaempferol-3-O-β-rutinoside (HP29), and cinchonain B (HP133) are particularly associated with the most effective group of extracts (group 3), highlighting their critical role in antifungal activity.

Conversely, some compounds showed negative Pearson correlation values, such as 2,4,6-trihydroxyacetophenone-2,4-di-O-β-D-glucopyranoside (HP58) with −0.626 and 1,5-O-dicaffeoylquinic acid (HP65) with −0.676. These negative correlations suggest that their presence may be associated with reduced antifungal efficacy, potentially interfering with the activity of bioactive compounds and playing a secondary role within the extract mixture.

The integration of Pearson correlation results, the phytochemical profile, the PLS-DA model, and the IC_50_ values reveals that total phenols, particularly condensed tannins such as procyanidins, are the primary determinants of the antifungal activity of the extracts. These metabolites proved a consistent and significant association with lower IC_50_ values, underscoring their substantial contribution to the inhibition of *Fo*. Conversely, while flavonoids showed negative correlations in the Pearson analysis, the PLS-DA model found compounds such as rutin (HP21), kaempferol-3-O-β-rutinoside (HP29), and quercetin-3-O-β-D-glucopyranoside (HP23) with high VIP values and strong positive correlations. This suggests that although flavonoids may not directly contribute to the antifungal effect, they could play a synergistic or modulatory role in combination with other bioactive metabolites responsible for the observed biological activity. In conclusion, condensed tannins, especially procyanidins, appear as the most consistently relevant metabolites across the analyses, positioning them as the key contributors to the antifungal activity of the extracts.

## 4. Discussion

The total extraction method was the most efficient regarding the extraction yield, with TML standing out (13.1%). However, although the total maceration method extracted more compounds, no enrichment of these with bioactive metabolites was observed. On the other hand, the CMML (8.5%) and UAML (11.4%) extracts showed lower yields, although they were more selective and effective in extracting bioactive metabolites.

Ultrasonication in UAML facilitates the rupture of cell walls by cavitation, promoting the release of intracellular compounds. This favors a more selective extraction of bioactive metabolites, such as phenols and flavonoids, at low temperatures and in less time. This protects the integrity of thermosensitive compounds and allows for a more enriched phytochemical profile than UAML [23,24]. On the other hand, the CM method facilitates the extraction of specific fractions at each stage, contributing to a higher concentration of key metabolites in the final extract [25]. Although this process results in a lower total yield, it maximizes the concentration of compounds related to the solvents used, reflected in a phytochemical profile enriched in tannins, flavonoids, and total phenols. Lezoul et al. demonstrated that using solvents of varying polarities allows for the extraction of bioactive compounds more selectively and efficiently in medicinal plants [26]. Their research shows that polar solvents favor the extraction of polyphenols and flavonoids, while less polar solvents can isolate other compounds. Together, these results highlight how the choice of extraction method impacts the extract yield and influences the amount and type of secondary metabolites extracted. Total maceration maximizes the yield but includes a more heterogeneous mixture, while sonication-assisted maceration and consecutive maceration optimize the extraction of specific and bioactive metabolites, achieving extracts more enriched in compounds of interest.

Some researchers have highlighted the antimicrobial potential of medicinal plants such as *Hamelia patens* Jacq. depends on the plant part used, the extraction method [27,28], the phytochemical composition, and the concentration of the extracts [29]. The presence of bioactive compounds, such as alkaloids, phenolic compounds, and terpenes, gives HP a significant antifungal effect derived from the synergistic action of these metabolites on different molecular targets [30,31,32]. Alkaloids inhibit protein synthesis and denature the fungal cell membrane, destabilizing the cell walls and causing cell death [33]. Flavonoids alter the structure of the fungal cell membrane, increasing permeability and resulting in the loss of essential ions and nutrients. Additionally, flavonoids disrupt polysaccharide synthesis, weakening membrane integrity and reducing resistance to environmental factors. They also inhibit enzymes involved in synthesizing key cell wall components, such as glucanases [34,35]. Phenolic acids induce oxidative stress, causing damage to membranes, proteins, and nucleic acids, compromising the viability of fungal cells, while polyphenols alter the permeability of membranes, causing the loss of cellular content and death. On the other hand, tannins can bind to proteins or other biomolecules, preventing the growth of microorganisms. They can also denature fungal proteins to reduce their pathogenic properties [36].

The identification of secondary metabolites in HP extracts coincides with previous reports. Flores-Sánchez et al. used nuclear magnetic resonance spectroscopy (NMR) and ultra-performance liquid chromatography (UPLC) to identify compounds such as rutin, kaempferol, and chlorogenic acid, highlighting the phytochemical diversity of this species [37]. Noor et al., in a comprehensive review, supported the presence of these metabolites in methanolic extracts of HP based on many studies that used methods such as HPLC-MS and GC-MS for their identification and quantification [10]. Paz et al. applied high-performance liquid chromatography coupled with electrospray ionization mass spectrometry (HPLC-ESI-MS) to characterize compounds such as catechin, epicatechin, and quercetin-3-O-β-D-glucopyranoside, allowing for a detailed analysis of the phenolic profile of the extracts [27]. Complementing these findings, Maamoun et al. identified, in addition to rutin and isoquercetin, the presence of soyasaponin Bb in *H. patens* leaves by UPLC/ITMS/MS [38]. Ahmad et al. reinforced this perspective by including compounds such as flavonoids, condensed tannins, and phenolic acids in their review, highlighting the antioxidant, anti-inflammatory, and antimicrobial activity associated with these metabolites [39]. For their part, Ríos and Aguilar-Guadarrama found indole and oxindole alkaloids (aricin and aricin oxindole), as well as flavonoids such as catechin, triterpenes (ursolic acid and rotundic acid), and sterols (stigmasterol and β-sitosterol) [40].

Several authors suggest that secondary metabolites in extracts can provide enhanced defense against pathogenic fungi through synergistic interactions [41,42,43]. Rutin exhibits antifungal potential by disrupting the cell membrane and inhibiting protein and nucleic acid synthesis [44]. Kaempferol induces oxidative stress in fungal cells and alters the cell wall structure [45]. Quercetin disrupts the plasma membrane by reducing ergosterol levels and induces apoptosis through the accumulation of reactive oxygen species (ROS) and mitochondrial dysfunction. Additionally, it inhibits nucleic acid and protein synthesis and impairs virulence factors, such as biofilm formation and cell adhesion [46]. Likewise, catechin and epicatechin have shown significant antifungal activity, especially in inhibiting fungal toxins such as aflatoxin B1 produced by *Aspergillus flavus*. These compounds act by reducing oxidative stress in fungal cells [47]. Phenolic acids such as gallic acid, caffeic acid, ferulic acid, cinnamic acid, *p*-coumaroyl-isocitric acid, caffeoyl-isocitric acid, *p*-coumaric acid, and caffeoylquinic acid also possess antifungal properties through different mechanisms of action, among which the most notable is the disruption of the cell membrane causing the leakage of cytoplasmic content and inhibiting the synthesis of glucans in the cell wall [48,49].

## 5. Conclusions

The methanolic extracts of *Hamelia patens* Jacq. demonstrated significant antifungal activity against *Fusarium oxysporum* f. sp. *radicis-lycopersici* primarily attributed to the presence of secondary metabolites such as tannins and phenolic acids. The results emphasize the critical influence of extraction methods and plant tissue selection on the diversity and concentration of bioactive compounds. CMML demonstrated the most effective antifungal activity, highlighting the importance of optimizing extraction techniques to maximize metabolite recovery and biological efficacy. Despite these advances, the specific mechanisms behind the synergistic effects of major compounds remain unclear, representing a key knowledge gap. Future research should focus on isolating and characterizing these metabolites to elucidate their antifungal mechanisms. Additionally, evaluating the scalability and economic feasibility of these extraction methods for agricultural applications is essential to position these extracts as viable alternatives for sustainable phytopathogen management.

## Figures and Tables

**Table 1:** Percentage yields of leaf and flower extracts of *H. patens*.

CMHL	CMDL	CMML	TML	UAML	CMHF	CMDF	CMMF	TMF	UAMF
0.8 ± 0.1 ^E^	1.2 ± 0.02 ^E^	8.5 ± 0.8 ^C^	13.1 ± 0.9 ^A^	11.4 ± 0.6 ^B^	0.2 ± 0.005 ^E^	0.6 ± 0.02 ^E^	4.4 ± 0.8 ^D^	4.8 ± 0.6 ^D^	4.6 ± 0.6 ^D^

Different letters indicate significant differences *p* < 0.05. Data are expressed as mean ± standard deviation, n = 4.

**Table 2:** Phytochemical profile of leaf and flower extracts of *H. patens*.

Metabolites	CMHL	CMDL	CMML	TML	UAML	CMHF	CMDF	CMMF	TMF	UAMF
TP	30.7 ± 0.1 ^J^	80.7 ± 0.6 ^G^	172.8 ± 0.5 ^C^	154.1 ± 0.4 ^D^	203.8 ± 0.4 ^A^	56.3 ± 0.4 ^H^	148.0 ± 0.7 ^E^	149.1 ± 0.4 ^I^	132.9 ± 0.7 ^F^	175.9 ± 0.6 ^B^
Fl	5.1 ± 0.6 ^F^	8.9 ± 0.6 ^E^	9.7 ± 0.1 ^D^	32.4 ± 0.2 ^A^	31.9 ± 0.2 ^A^	3.4 ± 0.2 ^F^	15.4 ± 0.5 ^C^	8.1 ± 0.4 ^E^	26.6 ± 0.3 ^B^	27.1 ± 0.4 ^B^
TC	ND	ND	47.3 ± 0.2 ^A^	30.5 ± 0.3 ^C^	39.4 ± 0.6 ^B^	ND	ND	26.1 ± 0.1 ^D^	19.2 ± 0.4 ^E^	25.8 ± 0.5 ^D^
S	168.3 ± 0.6 ^J^	585.5 ± 0.6 ^C^	544.2 ± 0.8 ^E^	464.6 ± 0.4 ^H^	967.5 ± 0.5 ^B^	214.7 ± 0.6 ^I^	475.4 ± 0.5 ^G^	576.2 ± 0.6 ^D^	493.7 ± 0.3 ^F^	1028.2 ± 0.6 ^A^
TA	ND	0.2 ± 0.03 ^D^	0.4 ± 0.02 ^B^	0.3 ± 0.04 ^C^	0.5 ± 0.02 ^A^	ND	0.1 ± 0.04 ^F^	0.2 ± 0.05 ^E^	0.1 ± 0.02 ^F^	0.3 ± 0.02 ^C^
TS	48.3 ± 0.3 ^C^	11.9 ± 0.2 ^E^	11.9 ± 0.2 ^G^	1.8 ± 0.4 ^H^	60.4 ± 0.4 ^A^	43 ± 0.2 ^D^	26.18 ± 0.3 ^F^	11.2 ± 0.2 ^G^	1.7 ± 0.2 ^H^	56.5 ± 0.2 ^B^

TP: Total phenols (meq-AG/g). Fl: Flavonoids (meq-Q/g). TC: Condensed tannins (meq-C/g). S: Saponins (meq-D/g). TA: Total alkaloids (meq-G/g). TS: Total sterols (meq-Ch/g). ND: Not detected. Different letters indicate significant differences (α = 0.05) in each row. Results are expressed as mean ± SD (n = 4).

**Table 3:** Percentage of the mean inhibitory concentration (IC_50_) of the methanolic extracts of *H*. *patens* on *F. oxysporum* f. sp. *radicis-lycopersici*.

Extract	CMML	UAML	TML	CMMF	UAMF	TMF
IC_50_	3.7 ± 0.3 ^A^	4.3 ± 0.15 ^AB^	4.5 ± 0.05 ^BC^	4.8 ± 0.3 ^BC^	4.9 ± 0.13 ^C^	5 ± 0.14 ^C^

Different letters indicate significant differences α = 0.05 between treatments. Data are expressed as mean ± standard deviation; n = 4.

**Table 4:** Pearson correlation between the phytochemical profile and the antifungal effect IC_50_ of the extracts.

Metabolites	TP	Fl	TC	S	TA	TS
IC_50_	0.735	−0.345	0.902	−0.133	0.686	−0.034

TP: Total phenols. Fl: Flavonoids. TC: Condensed tannins. S: Saponins. TA: Total alkaloids. TS: Total sterols.

**Table 5:** Identification and quantification of secondary metabolites in leaf and flower extracts of *H*. *patens*.

ID	NAME	RT	Formula	TMF	UAMF	CMMF	TML	UAML	CMML
Flavonoids									
HP1	Leucodelphinidin	0.8	C_15_H_14_O_8_	ND	ND	ND	2432	ND	ND
HP2	Epigallocatechin(4β,8)-gallocatechin	1.01	C_30_H_26_O_14_	ND	ND	ND	10760	ND	ND
HP3	Kushecarpins C	1.03	C_17_H_16_O_7_	ND	ND	ND	2264	ND	ND
HP4	Dihydromorin	1.39	C_15_H_12_O_7_	ND	ND	ND	2772	2203	ND
HP5	3-O-β-D-galactopyanosyl quercetin	1.41	C_21_H_22_O_12_	ND	ND	ND	ND	37575	33123
HP6	(+)-catechin	1.49	C_15_H_14_O_6_	26268	22055	28862	41223	34568	18759
HP7	3,6,7-trimethylquerce-tagetin	1.58	C_18_H_16_O_8_	ND	ND	ND	ND	ND	20495
HP8	Nevadensin-7-O-[α-L-rhamnosyl(1→6)]-β-D-glucoside	1.63	C_30_H_36_O_16_	62335	97856	ND	ND	ND	ND
HP9	Kaempferol-3-O-β-D-glucopyranosyl-(1→2)-β-D galactopyranosyl-(1→2)-β-D-glucopyranoside	1.77	C_33_H_40_O_21_	ND	ND	ND	119497	117472	132575
HP10	3-O-[β-D-glucopyranosyl-(1→2)]-β-D-23 glucopyranosyl-7-O-α-L-glucopyranosyl-kaempferol	1.93	C_33_H_40_O_20_	ND	ND	ND	67421	73224	ND
HP11	Leufolin A	2.09	C_30_H_36_O_12_	388894	338925	563157	1540233	1450132	1511028
HP12	Taxifolin	2.09	C_15_H_12_O_7_	ND	ND	ND	ND	ND	2220
HP13	(-)-epicatechin	2.1	C_15_H_14_O_6_	587022	533031	707757	1234805	1169347	1212186
HP14	Tectoruside	2.1	C_21_H_30_O_13_	ND	ND	ND	13624	12724	39772
HP15	7-α-L-rhamnosyl kaempferol 3-O-β-D-glucopyranosyl (1→6)-β-D-glucopyranoside	2.17	C_33_H_40_O_20_	ND	ND	ND	97367	98022	57842
HP16	3-epicaryoptin	2.48	C_26_H_36_O_9_	ND	17766	12728	ND	18309	8790
HP17	Safflomin C	2.58	C_30_H_30_O_14_	ND	ND	ND	8656	9212	6332
HP18	Cinerenin	2.78	C_17_H_20_O_6_	ND	ND	ND	ND	1470	2283
HP19	Taxifolin-3-O-glucoside	2.81	C_21_H_22_O_12_	ND	ND	ND	3393	ND	1875
HP20	Quercetin-3-O-β-D-xylopyranosyl (1→2)-β-D-glucopyranoside	2.92	C_26_H_28_O_16_	ND	ND	ND	8999	ND	ND
HP21	Rutin	3.24	C_27_H_30_O_16_	1709140	1672788	2174119	4556630	4457198	4797246
HP22	2′-Hydroxy-isoophiopogonone A	3.25	C_18_H_14_O_7_	ND	ND	ND	37517	44005	29642
HP23	Quercetin-3-O-β-D-glucopyranoside	3.34	C_21_H_20_O_12_	205137	196998	263207	610566	597554	755679
HP24	3,5,8-trihydroxy-3′,4′-dimethoxyflavone	3.35	C_17_H_14_O_7_	ND	ND	ND	ND	ND	1316
HP25	7-O-α-L-rhamnosyl-3-O-β-D-glucopyranosyl kaempferol	3.49	C_27_H_30_O_15_	167226	161174	257202	1729986	1717769	2053719
HP26	3,8-dihydroxy-4,10-dimethoxy-7-oxo-[2] benzopyrano [4,3-b] [1]benzopyran-7-(5H)-one	3.59	C_18_H_14_O_7_	ND	ND	ND	20651	22889	24191
HP27	Dichotomitin	3.65	C_18_H_14_O_8_	ND	ND	ND	ND	ND	11606
HP28	Apigenin-7-O-α-L-rhamnose(1→4)-6″-O-acetyl-β-D-glucoside	3.72	C_29_H_32_O_15_	ND	ND	ND	ND	ND	7685
HP29	Kaempferol-3-O-β-rutinoside	3.75	C_27_H_30_O_15_	151771	150657	290560	3098938	3132193	2931083
HP30	Polygalaxanthone III	3.93	C_25_H_24_O_12_	ND	ND	4547	ND	ND	ND
HP31	Quercitrin	3.93	C_21_H_20_O_11_	16538	13844	26559	283357	300008	253660
HP32	2′-hydroxyophiopogonone A	3.95	C_18_H_14_O_7_	ND	ND	ND	ND	14205	ND
HP33	2,5,7-trihydroxy-2-(4-hydroxyphenyl)-2H-chromen-3,4-dione	4	C_15_H_10_O7_8_	ND	ND	ND	3002	ND	2744
HP34	(−)-epicatechin-pentaacetate	4.5	C_25_H_24_O_11_	ND	ND	9723	4.38	29938	31823
HP35	[(2aS,4aS,5S,7bS)-5-(beta-D-glucopyranosyloxy)-1-6 oxo-2a,4a,5,7b-tetrahydro-1H-2,6-dioxacyclopenta[cdinden-4-yl]methyl 4-hydroxybenzoate	4.66	C_23_H_24_O_12_	ND	ND	ND	ND	7477	ND
HP36	5-hydroxyauranetin	4.88	C_20_H_20_O_8_	ND	ND	ND	ND	ND	20790
HP37	(+)-catechin-pentaacetate	4.93	C_25_H_24_O_11_	ND	ND	47958	16184	15846	7067
HP38	Kushenol B	5.05	C_30_H_36_O_6_	ND	ND	1985	ND	ND	ND
HP39	Mururin A	5.55	C_24_H_16_O_9_	14089	11815	ND	90205	97690	92059
HP40	Kaempferol	6.12	C_15_H_10_O_6_	ND	ND	ND	2238	2292	ND
HP41	Spinogenin C6	10.09	C_29_H_46_O_5_	17607	ND	ND	ND	ND	ND
HP42	Spinogenin C7	11.48	C_29_H_44_O_5_	259258	246481	ND	ND	ND	ND
Phenolic Acids									
HP43	Dihydroferulic acid 4-O-glucuronide	0.58	C_16_H_20_O_10_	ND	ND	ND	26664	29738	48766
HP44	Neochlorogenic acid	1	C_16_H_18_O_9_	131226	141099	211266	170057	179067	122738
HP45	3-O-trans-coumaroylquinic acid	1.37	C_16_H_18_O_8_	1695	1633	2426	ND	ND	2531
HP46	(1S,3R,4R,5R)-1,3,4-Trihydroxy-5-{[(2E)-3-(2,3,4 trihydroxyphenyl)-2-propenoyl]oxy}cyclohexanecarboxilic acid	1.49	C_16_H_18_O_10_	14075	13675	19019	17460	16768	17740
HP47	Chlorogenic acid	1.5	C_16_H_18_O_9_	1016330	985206	1179508	1606306	1586282	1645918
HP48	4-hydroxy-3-[(2E)-3-(4-hydroxy-3-methoxyphenyl)-2-propenoyl]-6-methyl-2H-pyran-2-one	1.64	C_16_H_14_O_6_	ND	ND	2462	ND	ND	2388
HP49	trans-caffeic acid	1.69	C_9_H_814_	5790	5611	ND	4839	4783	4612
HP50	Rubianic acid	1.71	C_25_H_26_O_13_	ND	ND	ND	14802	15039	14706
HP51	Ferulic acid 4-glucuronide	1.82	C_16_H_18_O_10_	ND	ND	1598	ND	1618	2895
HP52	4-O-caffeoylquinic acid	2.05	C_16_H_18_O_9_	116608	201560	374898	234043	226244	303991
HP53	p-coumaroylquinic acid	2.05	C_16_H_18_O_8_	50785	47956	58848	43505	53057	24198
HP54	2,4,5-trihydroxybenzaldehyde	2.17	C_25_H_24_O_12_	6117	ND	ND	ND	ND	ND
HP55	Glucosyringic acid	2.37	C_15_H_20_O_10_	9453	11246	3561	2219	ND	ND
HP56	Methyl-4-O-(4″-hydroxy-3″,5″-dimethoxybenzoyl) chlorogenate	2.41	C_26_H_28_O_13_	ND	ND	ND	9664	ND	10050
HP57	3′-methoxyniduloic acid	2.45	C_12_H_16_O_5_	1858	2879	ND	ND	ND	ND
HP58	2,4,6-trihydroxyacetophenone-2,4-di-O-β-D-glucopyranoside	2.49	C_20_H_28_O_14_	374781	359251	300985	426292	398361	196779
HP59	1-caffeoyl-4-deoxyquinic acid	2.63	C_16_H_18_O_8_	ND	ND	12868	17824	18830	22374
HP60	Methyl gallate	2.77	C_8_H_8_O_5_	ND	ND	1422	2165	2176	3979
HP61	5-O-feruloylquinic acid	2.91	C_17_H_20_O_9_	55947	35730	56352	1014702	957685	1255983
HP62	Ligustrosidic acid	3.11	C_26_H_32_O_13_	ND	ND	ND	14785	ND	ND
HP63	7-O-[4′-O-(3″,4″-dihydroxycinnamyl)-β-D- glucopyranosyl]-6-methoxycoumarin	3.89	C_25_H_24_O_12_	ND	ND	ND	345614	317476	222283
HP64	1,3-O-dicaffeoylquinic acid	3.9	C_25_H_24_O_12_	326723	289600	ND	ND	ND	ND
HP65	1,5-O-dicaffeoylquinic acid	4.42	C_25_H_24_O_12_	711690	654563	980428	575472	545190	461925
HP66	Methyl rosmarinate	4.57	C_19_H_18_O_8_	ND	ND	7021	ND	ND	ND
HP67	1,4-O-dicaffeoylquinic acid	5	C_25_H_24_O_12_	ND	ND	85660	ND	ND	ND
HP68	1-O-methyl-3,5-O-dicaffeoylquinic acid methyl ester	5.37	C_27_H_28_O_12_	ND	ND	ND	10886	11027	ND
HP69	Terminolic acid	8.88	C_30_H_48_O_6_	247054	279796	ND	ND	ND	ND
HP70	2-hydroxyesculentic acid	11.48	C_30_H_46_O_7_	140991	132086	ND	ND	ND	ND
Glycosylated phenols									
HP71	Mudanoside A	0.44	C_14_H_18_O_9_	ND	ND	ND	2800	2953	2623
HP72	Polygoacetophenoside	0.53	C_14_H_18_O_10_	3187	ND	ND	2526	2529	3123
HP73	6-O-galloyl-glucose	0.63	C_13_H_16_O_10_	2993	2982	427405	ND	ND	ND
HP74	Mudanoside B	0.7	C_18_H_24_O_14_	17581	19582	31182	ND	ND	ND
HP75	Koaburaside	0.73	C_14_H_20_O_9_	ND	ND	ND	1912	0	2705
HP76	Parasorboside	0.77	C_12_H_20_O_8_	2059	2092	3099	3815	3830	2443
HP77	Viscumneoside II	1.39	C_25_H_26_O_13_	ND	ND	4778	ND	87890	ND
HP78	Ulmoside	1.53	C_21_H_32_O_14_	ND	ND	1606	ND	ND	92625
HP79	1-O-(2-acetoxybenzoyl)-2-deoxy-β-D-arabino-hexopyranose	1.6	C_15_H_18_O_8_	ND	ND	11679	36150	33765	30081
HP80	Galloylpaeoniflorin	1.68	C_30_H_32_O_15_	ND	ND	ND	13465	ND	ND
HP81	Cnideoside B	1.76	C_18_H_22_O_10_	ND	ND	ND	ND	2376	2122
HP82	Taraxacoside	1.76	C_18_H_22_O_10_	ND	ND	ND	2142	ND	ND
HP83	2-methoxy-4-acetylphenyl-1-O-β-D-apiofuranosyl(1″→6′)-β-D-glucopyranoside	1.79	C_20_H_28_O_12_	ND	ND	ND	12274	13173	ND
HP84	Paeonolide	1.8	C_20_H_28_O_12_	ND	ND	ND	ND	ND	11571
HP85	6-O-feruloylglucose	1.89	C_16_H_20_O_9_	ND	ND	ND	39474	38066	54414
HP86	4-(β-D-mannopyranosyloxy)benzyl 2,3-dihydroxy-3-methylbutanoate	1.98	C_18_H_26_O_10_	25186	23340	28866	24072	24298	37127
HP87	Pinnatifinoside I	2.06	C_24_H_22_O_10_	12900	ND	20091	126212	174289	152216
HP88	Feroxin B	2.21	C_35_H_36_O_12_	ND	ND	ND	ND	ND	4691
HP89	Apiopaeonoside	2.22	C_20_H_28_O_12_	ND	ND	ND	21590	20597	ND
HP90	6′-O-galloyl-homoarbutin	2.3	C_20_H_22_O_11_	ND	ND	ND	ND	ND	1825
HP91	Polygalatenoside E	2.38	C_25_H_28_O_15_	ND	ND	ND	ND	ND	12181
HP92	4-methoxybenzal-dehyde-2-O-β-D-xylosyl(1→6)β-D-glucopyranoside	2.49	C_19_H_26_O_12_	13398	12921	10006	ND	17577	ND
HP93	6″-O-p-hydroxybenzoyliridin	2.59	C_21_H_30_O_15_	5597	7374	12856	29914	30647	27107
HP94	Decaffeoylacteoside	2.73	C_20_H_30_O_12_	ND	ND	ND	13770	13591	26603
HP95	Sibirioside A	2.85	C_21_H_28_O_12_	ND	ND	ND	2202	ND	ND
HP96	Helonioside B	2.9	C_34_H_40_O_18_	ND	ND	ND	180510	169261	302130
HP97	2-methoxy-4-acetylphenol 1-O-α-L-rhamnopyranosyl(1″→6′)-β-D-glucopyranoside	2.98	C_21_H_30_O_12_	ND	ND	ND	ND	1944	ND
HP98	Lucidumoside D	3.08	C_27_H_36_O_13_	ND	ND	ND	ND	5832	ND
HP99	Nitensoside B	3.86	C_28_H_32_O_16_	11136	10375	16117	ND	ND	ND
HP100	Ningposide C	3.95	C_17_H_20_O_8_	ND	ND	ND	1956	ND	2482
HP101	2,3,5,4′-tetrahydroxystilbene-2,3-O-β-D-glucopyranoside	4.14	C_26_H_32_O_14_	ND	ND	6484	11626	10530	ND
HP102	Linusitamarin	4.24	C_17_H_22_O_9_	ND	2120	2423	ND	ND	ND
HP103	Benzoylpaeoniflorin	4.35	C_30_H_32_O_12_	ND	ND	ND	ND	ND	2129
HP104	Glucopyranoside-4-benzoyloxy-2-buten	4.46	C_23_H_32_O_12_	ND	ND	3427	ND	ND	ND
HP105	Macranthion G	4.51	C_26_H_36_O_12_	ND	ND	27660	62085	66792	108873
HP106	Syringaresinol-mono-O-β-D-glucoside	4.84	C_30_H_40_O_12_	ND	ND	ND	2655	2658	3513
HP107	Macranthion F	5.04	C_26_H_36_O_12_	11223	9191	13638	117633	99673	75264
HP108	Macrophylloside A	5.05	C_24_H_28_O_11_	23972	21699	35517	ND	ND	ND
HP109	Yadanzigan	5.57	C_26_H_38_O_14_	ND	ND	9981	ND	ND	ND
HP110	2,3,5,4′-tetrahydroxystilbene-2-O-(6″-O-α-D-glucopyranosyl)-β-D-glucopyranoside	5.61	C_26_H_32_O_14_	12523	ND	10509	ND	ND	ND
HP111	Hookeroside D	6.04	C_43_H_70_O_18_	ND	ND	ND	ND	ND	49798
HP112	Picfeltarraegenin VII	9.74	C_30_H_46_O_7_	14666	16110	ND	ND	ND	ND
Monoterpenes									
HP113	Adoxosidic acid	1	C_16_H_24_O_10_	2044	ND	ND	2220	ND	2300
HP114	Hastatoside	1.08	C_17_H_24_O_11_	ND	ND	ND	ND	ND	2313
HP115	Swertiamarin	1.18	C_16_H_22_O_10_	2635	2613	ND	2622	2634	ND
HP116	Gentiopicroside	1.32	C_16_H_20_O_9_	1891	ND	2884	ND	ND	ND
HP117	Menthoside	1.59	C_36_H_36_O_17_	ND	ND	7131	125393	109799	108104
HP118	Monotropein	1.69	C_16_H_22_O_11_	179767	180377	246709	161415	156978	132514
HP119	6-O-acetyl shanzhiside methyl ester	1.98	C_19_H_28_O_12_	75280	70686	83932	75549	78300	121138
HP120	Cistanoside F	2.01	C_21_H_28_O_13_	1228	ND	ND	ND	ND	ND
HP121	6′-O-β-D-glucosyl gentiopicroside	2.09	C_22_H_30_O_14_	ND	ND	1443	ND	ND	ND
HP122	Secoxyloganin	2.21	C_17_H_24_O_11_	37081	36415	35220	ND	6780	7307
HP123	6′-O-β-D-glucosylsweroside	2.58	C_22_H_32_O_14_	ND	ND	ND	ND	ND	9984
HP124	Secologanic acid	2.65	C_16_H_22_O_10_	ND	ND	ND	1772	1797	1874
HP125	10-O-acetylgeniposidic acid	2.83	C_18_H_24_O_11_	ND	ND	952	ND	ND	ND
HP126	6-feruloyl catalpol	2.92	C_25_H_30_O_12_	ND	ND	ND	9661	11719	7790
HP127	7-O-methylmorroniside	4.34	C_18_H_28_O_11_	ND	10051	3875	ND	ND	ND
HP128	Asperuloside tetraacetate	5.52	C_26_H_30_O_15_	ND	ND	24792	8595	8768	10917
Tannins									
HP129	Procyanidin B1	1.84	C_30_H_26_O_12_	684732	774235	1269581	2177037	1996963	2146441
HP130	Procyanidin C1	1.85	C_45_H_38_O_18_	127530	148932	242234	336355	304415	329580
HP131	Arecatannin A1	2.43	C_45_H_38_O_17_	407964	481294	808760	948830	897487	910121
HP132	Procyanidin C2	2.71	C_45_H_38_O_18_	47074	56882	104038	216598	192174	182465
HP133	Cinchonain b	3.25	C_24_H_20_O_9_	77994	93593	76065	583904	691321	454993
HP134	Procyanidin B2	3.39	C_30_H_26_O_12_	147904	144148	232541	478620	441583	420416
HP135	Arecatannin B1	3.41	C_45_H_38_O_17_	62213	ND	102964	88282	74894	73907
HP136	Cinchonain Ia	4.83	C_24_H_20_O_9_	147349	169798	ND	1875750	2108024	1761902
HP137	Procyanidin A2	4.96	C_30_H_24_O_12_	41838	36830	60844	62706	58178	55606
Quinones									
HP138	(3S)-abruquinone F	0.45	C_19_H_20_O_8_	ND	ND	ND	2275	ND	ND
HP139	2-acetylemodin	2.1	C_17_H_12_O_6_	ND	ND	1754	2313	2040	2236
HP140	Acetylalkannin	3.3	C_18_H_18_O_6_	ND	ND	ND	ND	ND	20499
HP141	Emodin-8-O-(6′-O-acetyl)-β-D-glucoside	4.97	C_23_H_22_O_10_	12892	13065	13386	ND	ND	ND
Lignans									
HP142	Smiglanin	1.16	C_15_H_16_O_9_	ND	ND	ND	ND	ND	2040
HP143	Mulberrofuran P	3.6	C_24_H_32_O_9_	ND	ND	ND	8632	9843	ND
HP144	Kadsurenin K	4.17	C_20_H_22_O_5_	ND	ND	2291	2032	2628	2574
HP145	Silandrin	4.23	C_25_H_22_O_9_	ND	ND	ND	ND	ND	15333
Organic Acids									
HP146	Mucic acid	0.33	C_6_H_10_O_8_	ND	1152	ND	2265	2597	ND
HP147	Citric acid	0.37	C_6_H_8_O_7_	ND	ND	ND	17020	19767	36679
HP148	2-hydroxy-1,2,3-propane tricarboxylic acid-2-ethylester	0.43	C_12_H_8_O_7_	ND	ND	ND	1613	1435	2142
HP149	Elculentic acid	9.79	C_16_H_20_O_10_	240522	384989	ND	ND	ND	ND
Saponins									
HP150	Esculentagenin	8.67	C_31_H_46_O_8_	14303	ND	ND	ND	ND	ND
HP151	Platycodigenin	10.09	C_30_H_48_O_7_	12298	11537	ND	ND	ND	ND
Terpenoids									
HP152	Pseudolaric acid B O-β-D-glucopyranoside	3.56	C_29_H_38_O_13_	12795	13420	ND	ND	ND	ND
HP153	Nobilin C	3.86	C_18_H_22_O_6_	ND	ND	ND	ND	ND	2410
Triterpenes									
HP154	Amurenlactone A	2.59	C_17_H_20_O_9_	24186	24121	29164	73258	33531	65578
HP155	3β,4β,23-trihydroxy-24,30-dinorolean-12,20(29)-dien-28-oic acid	10.5	C_28_H_42_O_5_	9024	10312	ND	ND	ND	ND
Miscellaneous									
HP156	Isomaltose	0.3	C_12_H_22_O_11_	2195	1556	1557	ND	ND	ND
HP157	Methyl-β-D-fructofuranoside	0.3	C_6_H_12_O_6_	ND	ND	ND	3412	3240	2883
HP158	Scoparone	2.09	C_11_H_10_O_4_	ND	ND	ND	2005	ND	2008
HP159	Aloeresin G	2.38	C_29_H_30_O_10_	ND	ND	ND	ND	ND	7383
HP160	Reserpic acid	2.75	C_22_H_28_N_2_O_5_	29413	25562	26793	ND	ND	ND
HP161	cis-osthenone	4.88	C_14_H_12_O_4_	ND	ND	ND	ND	ND	2398

ND: Not detected. The values corresponding to the extracts are expressed in relative units based on the “detector counts” of the UPLC-MS analyzer.

**Table 6:** Compounds with variable importance (VIP Scores) greater than 0.9 and Pearson correlation in the PLS-DA model for the antifungal activity of *Hamelia patens* Jacq. extracts.

ID	Metabolite	VIP	PC
HP21	Rutin	4.2149	0.831
HP29	Kaempferol-3-O-β-rutinoside	4.13671	0.767
HP25	7-O-α-L-Rhamnosyl-3-O-β-D-glucopyranosyl kaempferol	3.4767	0.863
HP136	Cinchonain Ia	3.2695	0.739
HP58	2,4,6-Trihydroxyacetophenone-2,4-di-O-β-D-glucopyranoside	3.1164	−0.626
HP61	5-O-Feruloylquinic acid	2.8689	0.875
HP129	Procyanidin B1	2.7091	0.787
HP11	Leufolin A	2.5472	0.792
HP23	Quercetin-3-O-β-D-glucopyranoside	2.0521	0.905
HP133	Cinchonain b	1.9992	0.637
HP81	Cnideoside B	1.974	0.821
HP13	(-)-Epicatechin	1.9036	0.789
HP47	Chlorogenic acid	1.8785	0.821
HP96	Helonioside B	1.828	0.945
HP10	3-O-[β-D-Glucopyra-nosyl-(1→2)]-β-D-glucopyranosyl-7-O-α-L-glucopyranosyl-kaempferol	1.5005	0.149
HP65	1,5-O-Dicaffeoylquinic acid	1.4565	−0.676
HP111	Hookeroside D	1.4352	0.88
HP131	Arecatannin A1	1.4263	0.679
HP134	Procyanidin B2	1.3573	0.72
HP119	6-O-Acetyl shanzhiside methyl ester	1.3469	0.884
HP63	7-O-[4′-O-(3″,4″-)-β-D-glucopyranosyl]-6-methoxycoumarin	1.3412	0.617
HP31	Quercitrin	1.2747	0.736
HP105	Macranthion G	1.112	0.956
HP44	Neochlorogenic acid	1.0942	−0.29
HP5	3-O-β-D-Galacopyanosyl quercetin	1.0524	0.817
HP64	1,3-O-Dicaffeoylquinic acid	1.0416	−0.625
HP52	4-O-Caffeoylquinic acid	1.0397	0.382
HP149	Elculentic acid	1.0309	−0.597
HP34	(−)-Epicatechin-pentaacetate	1.0188	0.844
HP15	7-α-L- kaempferol 3-O-β-D-glucopyranosyl (1→6)-β-D-glucopyranoside	0.9755	0.577
HP69	Terminolic acid	0.9626	−0.622
HP107	Macranthion F	0.9579	0.599
HP132	Procyanidin C2	0.9523	0.699
HP130	Procyanidin C1	0.9515	0.757
HP42	Spinogenin C7	0.9445	−0.626
HP87	Pinnatifinoside I	0.9321	0.81
HP6	(+)-Catechin	0.9294	−0.215
HP14	Tectoruside	0.9283	0.982
HP36	5-Hydroxyauranetin	0.9273	0.88
HP140	Acetylalkannin	0.9208	0.88
HP7	3,6,7-Trimethylquerce-tagetin	0.9207	0.88

**PC:** Pearson correlation. **VIP:** Importance of the variables in the projection.

## Data Availability

The data presented in this study are available in article and Supplementary Material.

## Supplementary Materials

Chromatographic data of all extracts used in this study are available at the following link: https://www.mdpi.com/article/10.3390/metabo15010023/s1, Figure S1: UPLC-MS chromatogram of Hamelia patens TMF extract; Figure S2: UPLC-MS chromatogram of Hamelia patens TML extract; Figure S3: UPLC-MS chromatogram of Hamelia patens CMMF extract; Figure S4: UPLC-MS chromatogram of Hamelia patens CMML extract; Figure S5: UPLC-MS chromatogram of Hamelia patens UAMF extract; Figure S6: UPLC-MS chromatogram of Hamelia patens UAML extract.
