# Supplementary material for: Comparison of Secondary Metabolite Extraction Methods in Hamelia patens Jacq. and Their Inhibitory Effect on Fusarium oxysporum f. sp. radicis-lycopersici
Source: Metabolites. 2025 Jan 6;15(1):23. doi: 10.3390/metabo15010023 (PMC11767014; doi:10.3390/metabo15010023)
Supplement: Supplementary file 1 [file metabolites-15-00023-s001.zip › metabolites-3386186-supplementary.pdf]

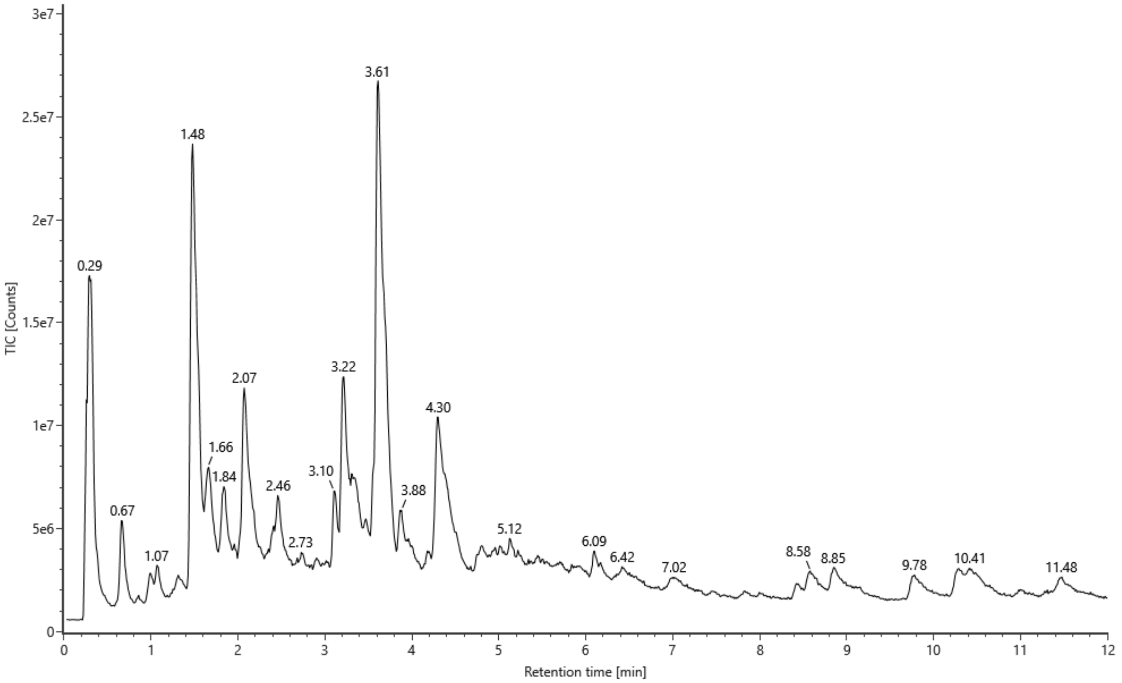

Figure S1. UPLC-MS chromatogram of *Hamelia patens* TMF extract

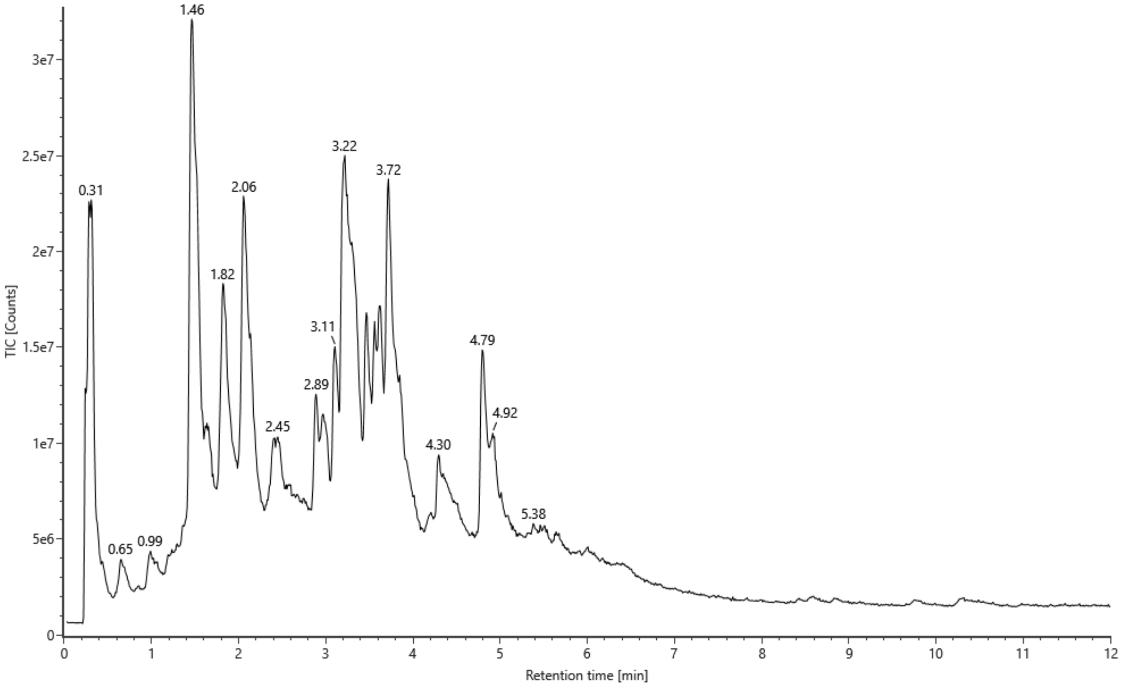

Figure S2. UPLC-MS chromatogram of *Hamelia patens* TML extract

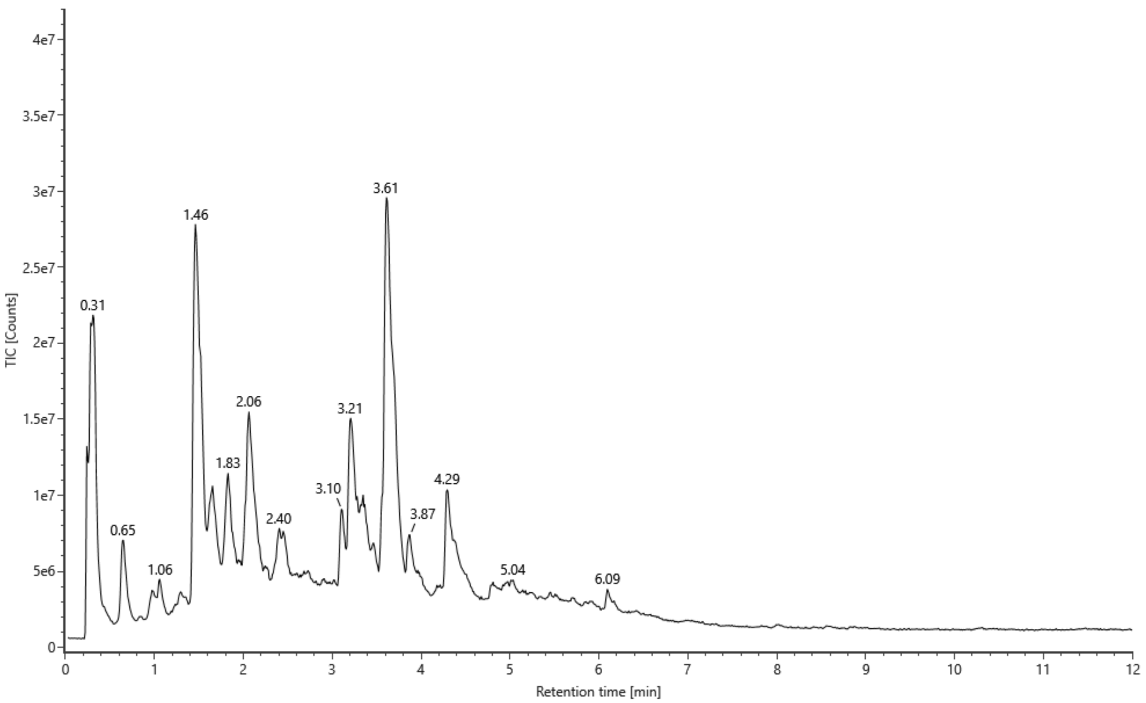

Figure S3. UPLC-MS chromatogram of *Hamelia patens* CMMF extract

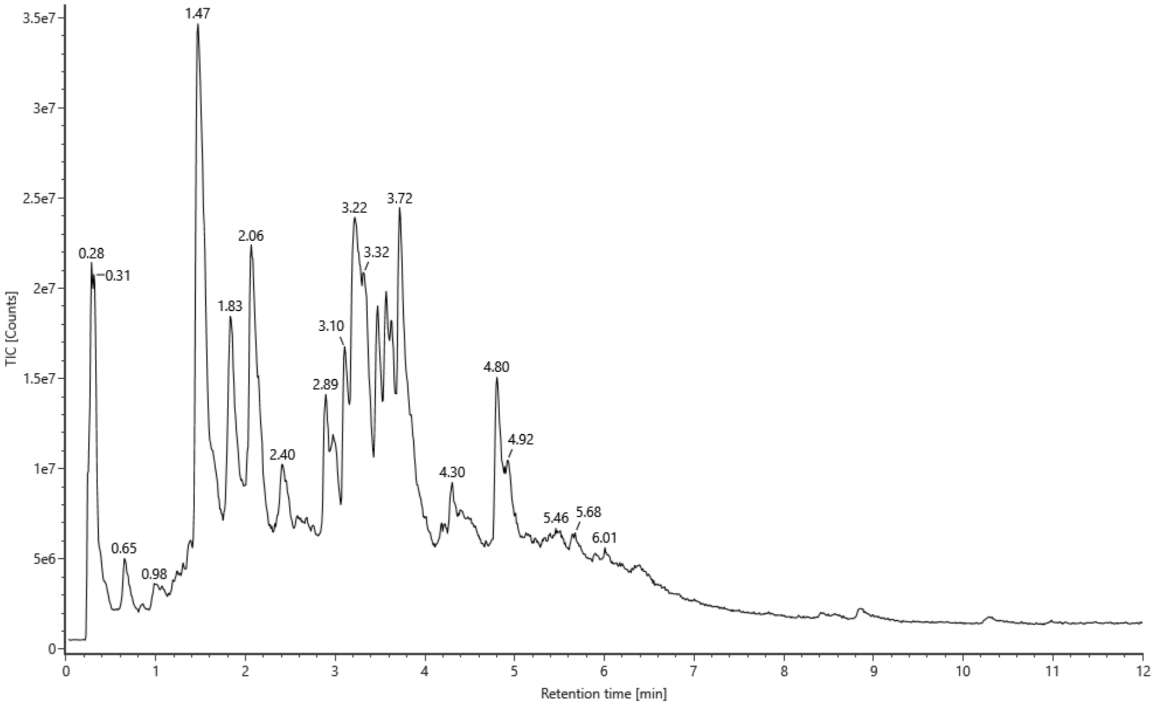

Figure S4. UPLC-MS chromatogram of *Hamelia patens* CMML extract

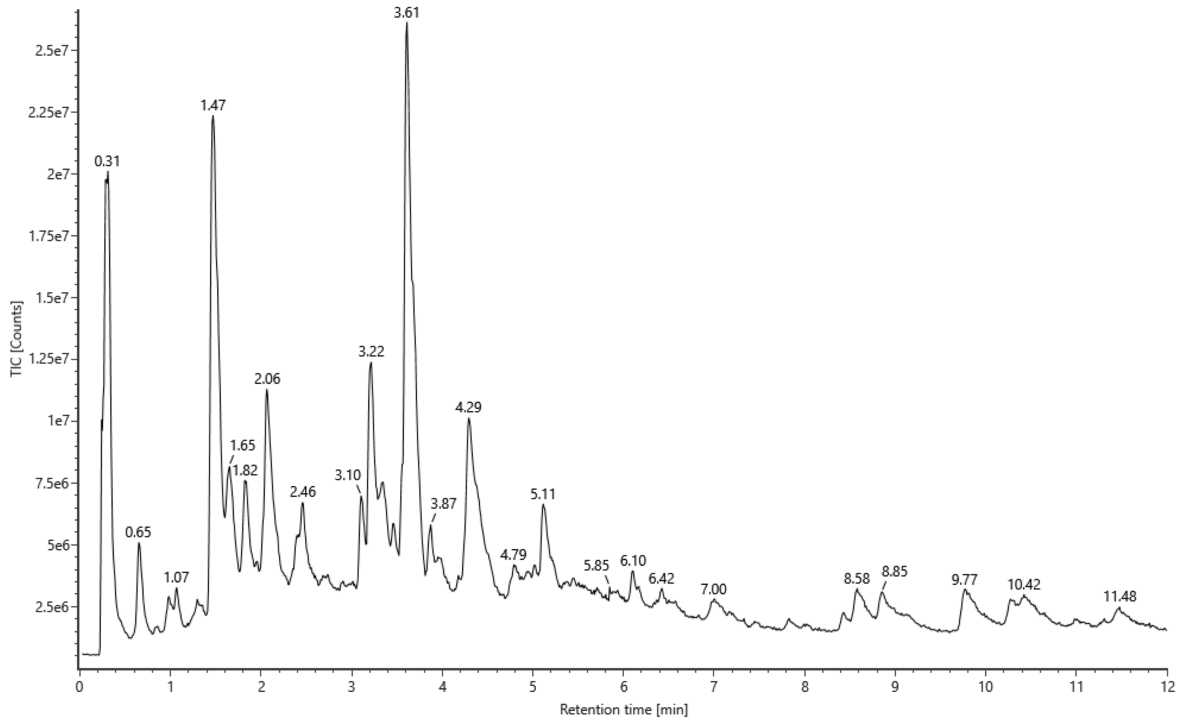

Figure S5. UPLC-MS chromatogram of *Hamelia patens* UAMF extract

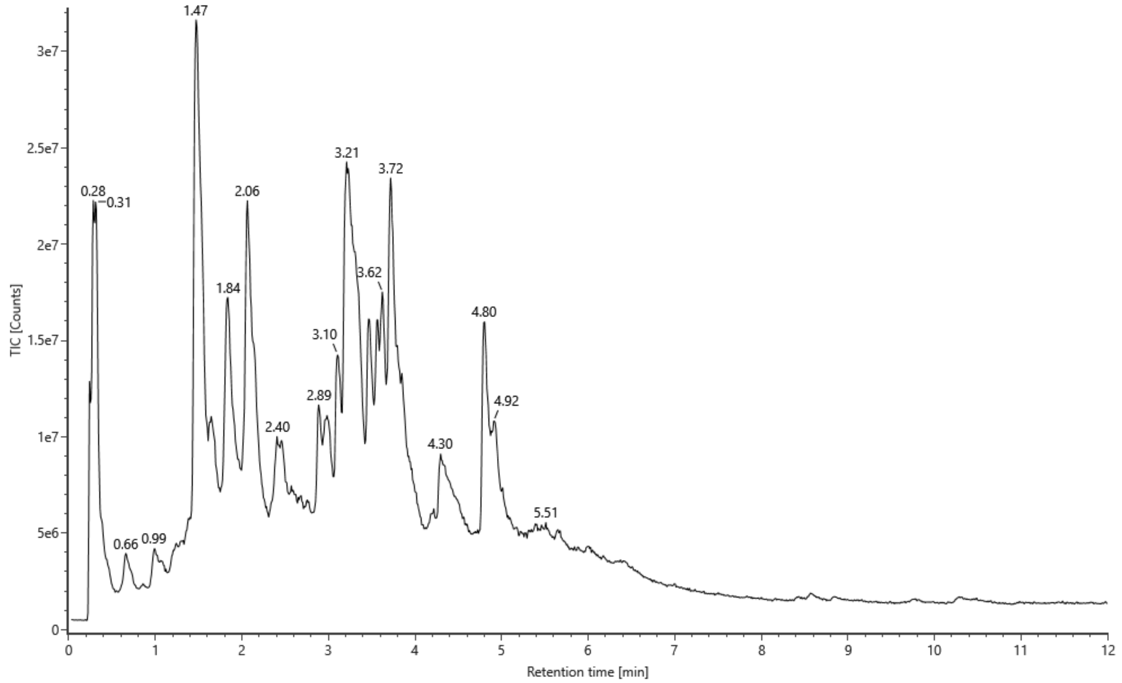

Figure S6. UPLC-MS chromatogram of *Hamelia patens* UAML extract
